# Rice straw increases microbial nitrogen fixation, bacterial and *nifH* genes abundance with the change of land use types

**DOI:** 10.3389/fmicb.2023.1283675

**Published:** 2024-02-28

**Authors:** Chen He, Kaikai Li, Jinku Li, Pingshan Fan, Yunze Ruan, Zhongjun Jia

**Affiliations:** ^1^Sanya Nanfan Research Institute of Hainan University, Hainan University, Sanya, China; ^2^School of Tropical Agriculture and Forestry, Hainan University, Haikou, China; ^3^Hainan Research Academy of Environmental Sciences, Haikou, China; ^4^State Key Laboratory of Black Soils Conservation and Utilization, Northeast Institute of Geography and Agroecology, Chinese Academy of Sciences, Changchun, China; ^5^Institute of Soil Science, Chinese Academy of Sciences, Nanjing, China

**Keywords:** carbon and nitrogen addition, nitrogen fixation, bacterial, *nifH* genes, soil utilization

## Abstract

Soil microorganisms play an important role in soil ecosystems as the main decomposers of carbon and nitrogen. They have an indispensable impact on soil health, and any alterations in the levels of organic carbon and inorganic nitrogen can significantly affect soil chemical properties and microbial community composition. Previous studies have focused on the effects of carbon and nitrogen addition on a single type of soil, but the response of soil microorganisms to varying carbon and nitrogen inputs under different land soil use types have been relatively understudied, leaving a gap in our understanding of the key influencing factors. To address this gap, we conducted a study in the tropical regions of Hainan province, focusing on four distinct land use types: natural forest soil (NS), healthy banana soil (HS), diseased banana garden soil (DS), and paddy soil (PS). Within each of these environments, we implemented five treatments: CK, RS (rice straw), RSN (rice straw and NH_4_NO_3_), RR (rice root), and RRN (rice root and NH_4_NO_3_). Our aim was to investigate how soil bacteria response to changes in carbon and nitrogen inputs, and to assess their potential for biological nitrogen fixation. The results showed that the addition of rice straw increased the absorption and utilization of nitrate nitrogen by microorganisms. The addition of rice roots (RR) did not increase the absorption capacity of inorganic nitrogen by microorganisms, but increased the content of poorly soluble organic carbon. Most importantly, the addition of rice straw increased microbial respiration and the utilization efficiency of N_2_ by microorganisms, and the further addition of ammonium nitrate increased microbial respiration intensity. With the change of soil type, the rice straw increases microbial nitrogen fixation, bacterial and *nifH* genes abundance. Meanwhile, microbial respiration intensity is an important factor influencing the differences in the structure of bacterial communities. The addition of inorganic nitrogen resulted in ammonium nitrogen accumulation, reduced microbial richness and diversity, consequently diminishing the soil microorganisms to resist the environment. Therefore, we believe that with the change of soil types, corresponding soil nutrient retention strategies should be devised and incorporated while reducing the application of ammonium nitrogen, thus ensuring healthy soil development.

## 1 Introduction

Soils are a complex substrate with the highest biodiversity on Earth (Wagg et al., 2019), and forms the largest activated land carbon pool (Crowther et al., 2016; Zhou et al., 2020). It is well-known that land use changes drive global change and affect the carbon and nitrogen cycles at the ecosystem level (Li et al., 2016). The dynamics of soil carbon and nitrogen under changes in different soil types are important for illustrating carbon and nitrogen cycle mechanisms and have thus been a focus of research (Guo and Gifford, 2002). The massive addition of chemical fertilizers by the agriculture industry has posed substantial environmental risks, including by serious damage to soil chemical properties (Idkowiak, 2004), resulting in a significant increase in emissions of greenhouse gas emissions (Zhang et al., 2013), nutrient damage, and disturbances of the soil microbial communities (Postma-Blaauw et al., 2010; Qiu et al., 2016).

Soil microbes drive the soil to achieve functions that facilitate soil carbon and nitrogen nutrient cycling, organic matter decomposition, transformation, as well as help suppress soil-borne diseases (Philippot et al., 2013; Ling et al., 2014; Finzi et al., 2015; Pieterse et al., 2016). It is well known that soils in the tropics are one of the essential terrestrial carbon pools globally (Jobbágy and Jackson, 2000), in which soil microorganisms play a key role in adjusting net soil carbon storage by mineralization of plant residues and soil organic matter. Previous work has found that nitrogen fertilization can have direct or indirect impact on soil microorganisms by introducing nutrients and altering soil physicochemical properties (Geisseler and Scow, 2014; Aparna et al., 2016). Current research suggests high correlations between changes in soil microbial community, land utilization, or management way and microbial-induced eco-system functions (Colman and Schimel, 2013; Bonner et al., 2018). As is well known, soil bacterial communities are important in biogeochemical cycle (Jenkins et al., 2017), and because soil bacteria respond rapidly to changes under the soil condition, it is often served as early bio-indicators of altered soil quality (Chen et al., 2017). Knorr et al. (2005) propose that nitrogen addition may potentially either promote or impede litter decomposition, a phenomenon seemingly tied to the nitrogen content accessible to plants (Hobbie, 2015). Nitrogen addition significantly changed the relative abundance of bacteria and fungi. The impact on soil microorganisms was subject to numerous factors, including differences in nitrogen addition time, soil hydrothermal conditions, elevation gradients, and vegetation types, as observed in different studies (Xu et al., 2021). Therefore, we can utilize high-throughput sequencing technology, which provides rough information about the entire microbial biodiversity in the background of complex environments and different farming practices (Bowles et al., 2014; Fierer, 2017; Beaudry et al., 2021).

Microorganisms also serve as the major decomposers of plant material because of their specific capacity to produce a large number of enzymes that degrade simple molecules (e.g., cellulose) and more complex plant-derived compounds (e.g., lignin) (Romaní et al., 2006). Soil microbes transform and utilize a variety of soil carbon substrates, take up carbon into their biomass, stabilize carbon, and emit CO_2_ in the soil (Bardgett et al., 2008; Wei et al., 2018). In agricultural production, straw addition is one of the main approaches for conventional exogenous carbon addition, and its decomposition and transformation involve a complex, long-term process of microbial community activity (Pausch and Kuzyakov, 2012). Crop straw, as a natural raw material, is rich in N, P, K and many other essential elements for plant growth. The addition of crop residues to the field is an important way to promote soil fertility and improve crop growth, which is important for the development of sustainable agriculture (Witt et al., 2000; Song et al., 2015). A large body of evidence shows that different characteristics ofcrop residues (straw) and environment factors can alter soil microbial communities’ diversity (Feng and Simpson, 2009; Merilä et al., 2010). Ma et al. found a significant positive effect of straw addition on soil microbial biomass, activity, and communities structure (Yu et al., 2016; Ma et al., 2019), resulting in an increase in soil grain structure and water-stabilized agglomerate content, which improves soil nutrients and water containment (Yin et al., 2015; Fierer, 2017). Other research found a significant increase in biomass of bacteria and actinomycete with short-term additions of crop straw to fields in Jiang Yan, China (Chen et al., 2017). The addition of carbon sources can indeed influence the physical and chemical properties as well as the microbial activity of farmland soil. It has been found that the incorporation of straw addition can improve soil structure, rendering it loser and more porous, while also increasing microbial and enzyme activities in the soil (Eagle et al., 2000; Sapkota, 2012). Biological nitrogen fixation stands as the second-largest contributor to soil nitrogen, trailing only behind mineral nitrogen, comprising approximately 16% of the global nitrogen input (Ollivier et al., 2011). The introduction of nitrogen, however, reduces the abundance and diversity of nitrogen-fixing microorganisms, with nitrate nitrogen emerging as one of the main influencing factors (Qin, 2021). The available nitrogen content in the soil showed a negative correlation with the community structure and abundance of nitrogen-fixing microorganisms. Furthermore, the addition of nitrogen fertilizer inhibited the growth of nitrogen-fixing microorganisms, resulting in a decrease in the diversity of nitrogen-fixing microorganisms in the soil (Hou et al., 2014). However, studies have shown that appropriate nitrogen fertilizer addition can increase the content of organic carbon in soil and increase the abundance of nitrogen-fixing bacteria, thereby promoting the nitrogen fixation capability of soil nitrogen-fixing microorganisms (Orr et al., 2012). Additional studies have also indicated that the abundance and community of nitrogen-fixing bacteria did not exhibit significant differences from the control group following nitrogen fertilization (Ogilvie et al., 2008; Berthrong et al., 2014). Therefore, exploring different types of soils assumes great significance in elucidating biological nitrogen fixation mechanisms. In conclusion, we propose several hypotheses, (1) Carbon and nitrogen supplementation alters the soil chemistry and bacterial composition of different utilization types; (2) Differences exist in the effects of different types of organic carbon (such as rice straw and roots) on soil properties and microorganisms, and the addition of organic carbon (straw) improves the nitrogen fixation capacity of organisms. Through this study, we aim to clarify the differences in bacterial responses to carbon and nitrogen within diverse land use types and to explore the potential of biological nitrogen fixation.

## 2 Materials and methods

### 2.1 Study sites

Soil samples were collected from four different locations as shown in Supplementary Figure S1: natural forest soil (NS), healthy banana soil (HS), diseased banana soil (DS), and paddy soil (PS). All four sample locations were adjacent to each other, and healthy banana soil, diseased banana soil, and paddy soil all developed from natural forest soil in the same location. The soil used is characterized as lateritic soil that developed from basalt parent material. All four soil samples were collected from Chengmai County, Hainan Province, China (19°23′, 110°15′). These soil samples were collected from the top 20 cm of soil at four randomly chosen locations. Each soil type was passed through a 2 mm sieve. A portion of the soil sample was dried in the laboratory for soil physicochemical property analysis, while the remainder was stored at −80°C for DNA extraction.

### 2.2 Experimental design

The experimental design encompassed the analysis of soil chemical properties across four distinct land use practices: natural forest soil, healthy banana soil, diseased banana soil, and paddy soil (Table 1). For each sample, 10 g soil was added into 120 mL culture flasks. These soil samples were then supplemented with ammonium nitrate (1 mg/g soil), rice straw and roots (0.15 g/pot), and ^15^N_2_ (^15^N_2_: O_2_: Ar = 20:20:60). Subsequently, the samples were incubated for a duration of 21 days under controlled conditions of 28°C and 60% of field water capacity. The experiment was organized into five treatment types, each with three replicates: control (CK), rice straw (RS), rice straw and ammonium nitrate (RSN), rice roots (RR) and ammonium nitrate (RRN) in Supplementary Figure S2. Gas collection was conducted at seven-day intervals to calculate cumulative CO_2_ emissions, and fresh culture gas was replenished as needed to maintain anaerobic during collection. Upon concluding the 21-day incubation period, various measurements were taken to assess the following: microbial carbon dioxide cumulative emissions, soil inorganic nitrogen content, soil insoluble organic C and N content, bacterial gene copy number, and bacterial high-throughput sequencing. These measurements were carried out to investigate the mechanisms of C and N utilization as well as the composition of soil bacterial communities across different soil types.

**Table 1:** Physical and chemical analyses of soil and organic materials.

Parameter	pH	Wc (%)	Vw (g/cm^3^)	TC (g/kg)	TN (g/kg)	NH_4_^+^ (mg/kg)	NO_3_^−^ (mg/kg)	C/N
NS	5.92	44.04	1.32	20.17	1.78	15.88	8.76	11.34
HS	5.14	47.00	1.28	18.90	1.54	11.30	13.50	12.27
DS	4.91	51.63	1.20	18.93	1.49	19.16	26.11	12.67
PS	5.32	47.40	1.25	19.12	1.57	18.67	18.67	12.22
Rice straw	–	–	–	383.32	5.22	–	–	73.39
Rice roots	–	–	–	399.65	8.68	–	–	46.07

NS, Natural forest soil; HS, Healthy banana soil; DS, Diseased banana soil; PS, Paddy soil. Wc, Water content; Vw, volume weight; TC, total C; TN, total N; NO3−, nitrate-N; NH4+, ammonium-N; C/N, TC/TN.

### 2.3 Physical and chemical analyses

All soil chemical properties were analyzed by the method of Lu (2000). Soil pH was determined using a soil-to-water ratio of 1:4 (w/v). Soil bulk weight and moisture content were determined via the drying method. Soil organic carbon (SOC) was measured using the K_2_Cr_2_O_7_ oxidation method. Soil total nitrogen (TN) was measured using the Kjeldahl method. Soil ammonium nitrogen (NH_4_^+^-N) and nitrate (NO_3_^−^-N) were measured using a continuous flow analyzer (AA3). Soil ^15^N abundances were measured using an isotope mass spectrometer (Germany). Soil microbial respiration was measured by closed incubation and CO_2_ emissions were determined by gas chromatography (Wang et al., 2017).

### 2.4 Microbial analyses

The total DNA of the soil microbial genome was extracted by using the Fast DNA® Spin Kit for Soil (MP Biomedicals) following the manufacturer’s instructions. PCR amplification was performed using 515F/907R (GTGCCAGCMGCCGCGG; CCGTCAATTCMTTTRAGTTT) universal primers, and sequencing was performed using the Illumina NovaSeq platform from Beijing Novogene Co, Ltd. The raw sequence data were demultiplexed and filtered using QIIME quality filters. The reads were truncated at any position with >3 consecutive quality scores ≤25. Sequences ≤200 bp were discarded before further analysis. Chimeras sequences were detected using a *de novo* algorithm. The trimmed sequences were clustered into operational taxonomic units (OTUs) at a 97% similarity cutoff, and the representative sequences were selected to annotate taxonomic information.

### 2.5 Statistical analyses

SPSS 24.0 software was used for the statistical analysis. One-way analysis of variance (ANOVA) and least significant difference (LSD) tests (*p* < 0.05) were used for multiple comparison analyses. All Graphs were generated by using Origin 2023 soft in this paper. The *vegan* R package was used to perform principal coordinate analysis (PCoA) based on Bray-Curtis distance and permutational multivariate analysis of variance. Redundancy analysis (RDA) was conducted to explore the relationships between soil chemical properties and bacterial communities. Network analysis was used to express the correlation and correlation degree between different OTUs. To reduce complexity, only OTUs with an average relative abundance >0.01 were retained to construct the network. Topological properties were manipulated, and visualization of the correlation network was achieved using R (4.2.3).

## 3 Results

### 3.1 Microbial respiration and inorganic N content

**Figure 1:**
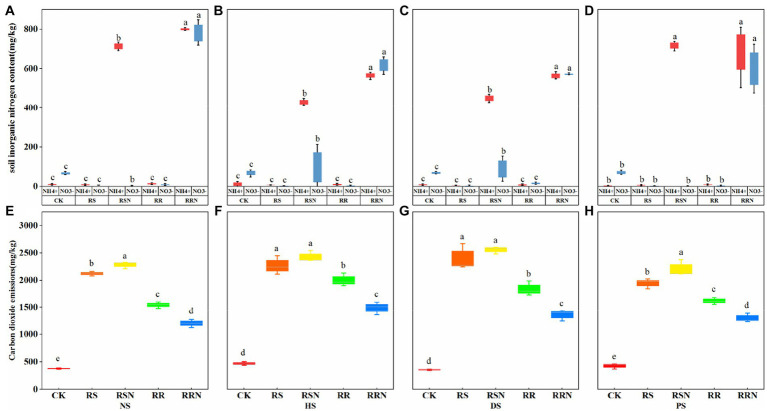
Inorganic N (NH_4_^+^, NO_3_^−^) content in natural forest soil **(A)**, healthy banana soil **(B)**, diseased banana soil **(C)**, paddy soil **(D)**. Microbial respiration (carbon dioxide emissions) in natural forest soil **(E)**, healthy banana soil **(F)**, diseased banana soil **(G)**, paddy soil **(H)** after cultivation for 24 days. Different letters indicate significant differences across different treatments (*p* < 0.05). Treatments: CK, no glucose and NH_4_NO_3_; RS, rice straw; RSN, rice straw and NH_4_NO_3_; RR, rice root; RRN, rice root and NH_4_NO_3_.

Rice straw and roots were the main inputs to soil C, and ammonium nitrate was the main N source to provide easily absorbed nutrients for microbial growth. In the RRN treatment, the inorganic N content was significantly higher than that in the other treatments as the soil types changed, and the ammonium nitrogen content was significantly higher than nitrate N in the RSN treatment. This indicates that the addition of rice straw increased the uptake and utilization of nitrate N by microorganisms, whereas the addition of rice roots did not increase microbial inorganic N uptake capacity (Figures 1A–D). In soils with different nitrogen disturbances, the addition of rice straw (RS, RSN) increased microbial respiration, while the addition of ammonium nitrate increased respiration intensity (Figures 1E–H).

### 3.2 Insoluble organic carbon and nitrogen content

**Figure 2:**
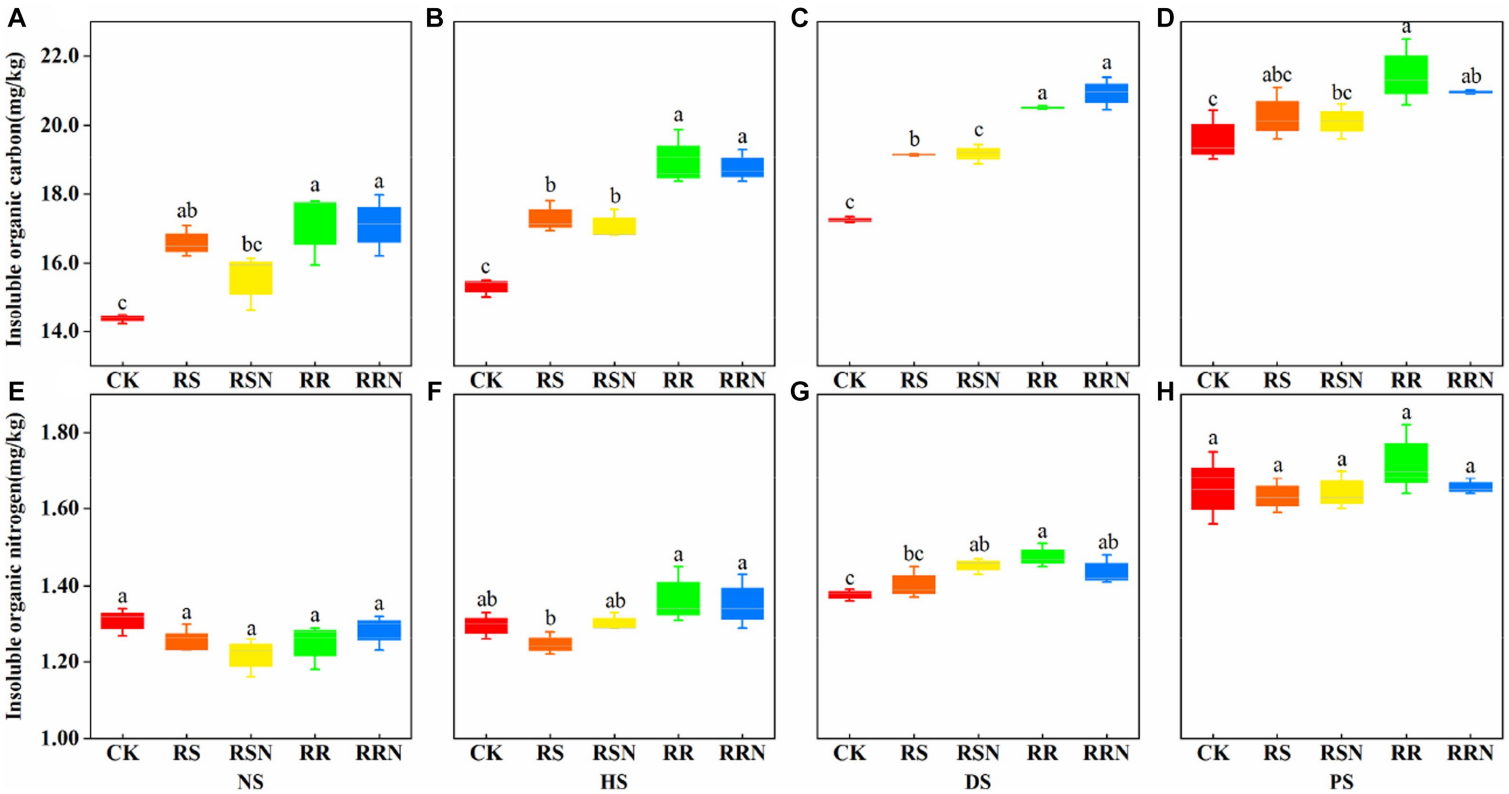
Insoluble organic C in natural forest soil **(A)**, healthy banana soil **(B)**, diseased banana soil **(C)**, paddy soil **(D)**. Insoluble organic N in natural forest soil **(E)**, healthy banana soil **(F)**, diseased banana soil **(G)**, paddy soil **(H)**. Different letters indicate significant differences between different treatments (*p* < 0.05). Treatments: CK, no glucose and NH_4_NO_3_; RS, rice straw; RSN, rice straw and NH_4_NO_3_; RR, rice root; RRN, rice root and NH_4_NO_3_.

We found that the addition of rice straw and rice roots increased insoluble organic carbon contents compared with the control, under different soil types, the content of insoluble organic carbon showed an obvious increasing trend (Figures 2A–D). This indicates that soil microorganisms are more inclined to use the root system when using exogenous C (rice roots), and the relatively low root C/N may be the main reason for the utilization of the root system. There was not significant difference in poorly soluble organic nitrogen content between NS and PS (Figures 2E,H), while organic nitrogen was significantly higher in the rice root treatment than in CK in DS and HS (Figures 2F,G). In NS and PS, the addition of rice roots had little effect on the insoluble organic nitrogen content, but there were differences between treatments in banana soil, indicating that different soil types affected the production of organic N.

### 3.3 ^15^N abundance

**Figure 3:**
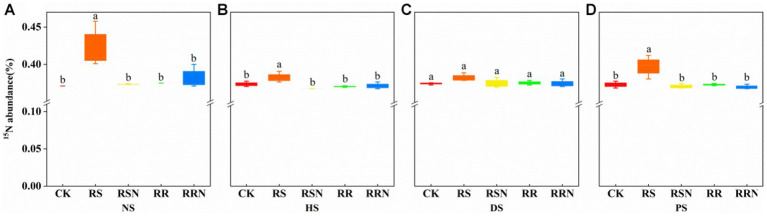
^15^N abundance in natural forest soil **(A)**, healthy banana soil **(B)**, diseased banana soil **(C)**, paddy soil **(D)**. Different letters indicate significant differences across different treatments (*p* < 0.05). Treatments: CK, no glucose and NH_4_NO_3_; RS, rice straw; RSN, rice straw and NH_4_NO_3_; RR, rice root; RRN, rice root and NH_4_NO_3_.

The addition of ^15^N_2_ was associated with a significant increase in ^15^N abundance in the RS treatment in NS, HS and PS compared to other treatments (Figures 3A,B,D), while there was no significant difference in the ^15^N abundance between treatment groups in DS (Figure 3C). This indicates that the addition of rice straw could increase the uptake and transformation of N_2_ by soil microorganisms and increase N conversion efficiency.

### 3.4 Copy number of 16S rRNA and *nifH* genes

**Figure 4:**
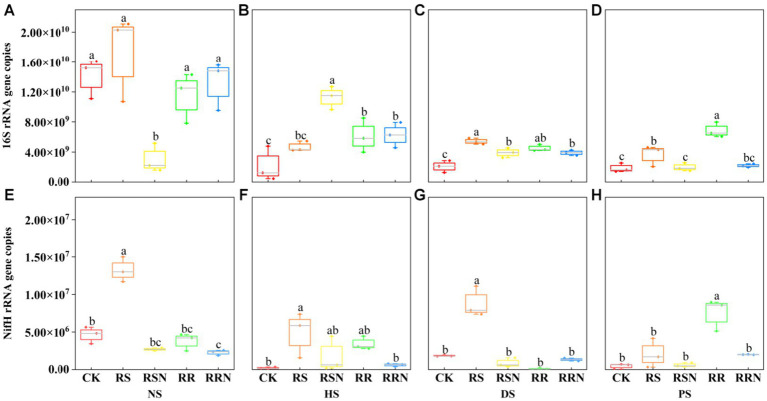
16S gene copy number in natural forest soil **(A)**, healthy banana soil **(B)**, diseased banana soil **(C)**, paddy soil **(D)**, *nifH* gene copy number in natural forest soil **(E)**, healthy banana soil **(F)**, diseased banana soil **(G)**, paddy soil **(H)**. Different letters indicate significant differences between different treatments (*p* < 0.05). Treatments: CK, no glucose and NH_4_NO_3_; RS, rice straw; RSN, rice straw and NH_4_NO_3_; RR, rice root; RRN, rice root and NH_4_NO_3_.

We found that the bacterial copy number was significantly higher in NS compared to the other soil types. However, under the RSN treatment, the bacterial copy number in NS decreased significantly (Figure 4A). In contrast, the copy number was higher in the RSN treatment in HS relative to other treatment groups (Figure 4B). In DS, the copy number of each treated bacteria was higher than in the control (Figure 4C), especially under RS treatment. In PS, the copy number of RR-treated bacteria was significantly higher than in other treatments (Figure 4D). The number of soil bacteria might decrease the changes in soil types, leading to an altered distribution of soil microorganisms. Nevertheless, the addition of rice straw and N could increase the number of bacteria. In natural forest soil and banana soil, the addition of rice straw can increase the copy number of nitrogen-fixing microorganisms (Figures 4E–G), but in rice soil, the addition of rice roots can significantly increase the nitrogen-fixing microbial copy number (Figure 4H).

### 3.5 Bacterial alpha diversity and bacterial community structure

Alpha diversity is expressed by four indexes: Chao1, ACE, Shannon and Simpson. In the Chao 1 and ACE indices, larger values indicate greater species richness (i.e., the greater the number of species in the community). Larger values for the Simpson and Shannon indices indicate higher community diversity (i.e., greater uniformity in the distribution of individuals). Here, we mainly used the Chao 1 and Shannon indices. The Chao 1 index value for the RSN treatment was significantly lower than values for other treatments (Supplementary Figures S3A,B) in both NS and HS, indicating reduced species richness. In DS, the value was significantly higher under the RR treatment (Supplementary Figure S3C), and, importantly, in PS, values for each treatment were significantly lower than in CK (Supplementary Figure S3D). In NS and HS, Shannon index values were significantly lower in RSN than in other treatments (Supplementary Figures S3E,F), while in DS, it was significantly higher under RR than under other treatments (Supplementary Figure S3G). Importantly, in PS, values were significantly lower in each treatment group than in CK (Supplementary Figure S3H), indicating reduced community diversity.

**Figure 5:**
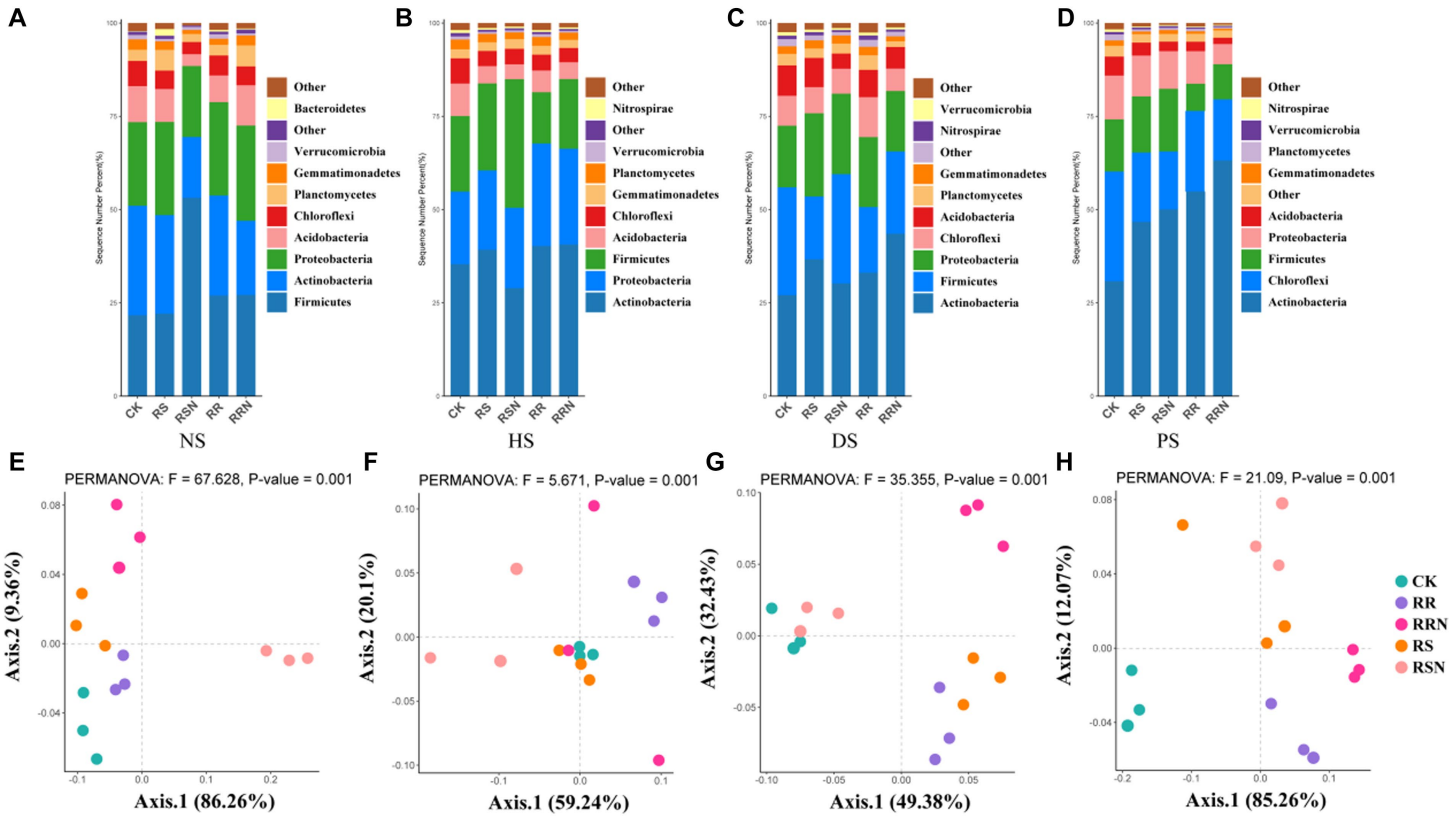
Community structure of bacteria in natural forest soil **(A)**, healthy banana soil **(B)**, diseased banana soil **(C)**, paddy soil **(D)**; PCoA of bacteria community structure in natural forest soil **(E)**, healthy banana soil **(F)**, diseased banana soil **(G)**, paddy soil **(H)**. Different letters indicate significant differences across different treatments (p < 0.05). Treatments: CK, no glucose and NH_4_NO_3_; RS, rice straw; RSN, rice straw and NH_4_NO_3_; RR, rice root; RRN, rice root and NH_4_NO_3_.

Bacterial community composition at the phylum level in NS differed considerably from other soil types (Figures 5A–D). In NS, the relative abundance of Firmicutes was significantly higher under the RSN treatment compared to other treatments (Figure 5A). In HS and DS, the relative abundance of Actinobacteria was the highest, but it was lower under the RSN treatment was lower than in CK (Figures 5B,C). The relative abundance of Actinobacteria was significantly higher in PS than in the other soil types (Figure 5D). PCoA revealed distinct separation of the RSN treatment from other treatments in NS (Figure 5E). In HS and DS, the degree of separation between each treatment decreased (Figures 5F,G), while in PS, the CK treatment showed significantly separation from the others (Figure 5H).

### 3.6 Relationships between bacterial community composition and soil properties

**Figure 6:**
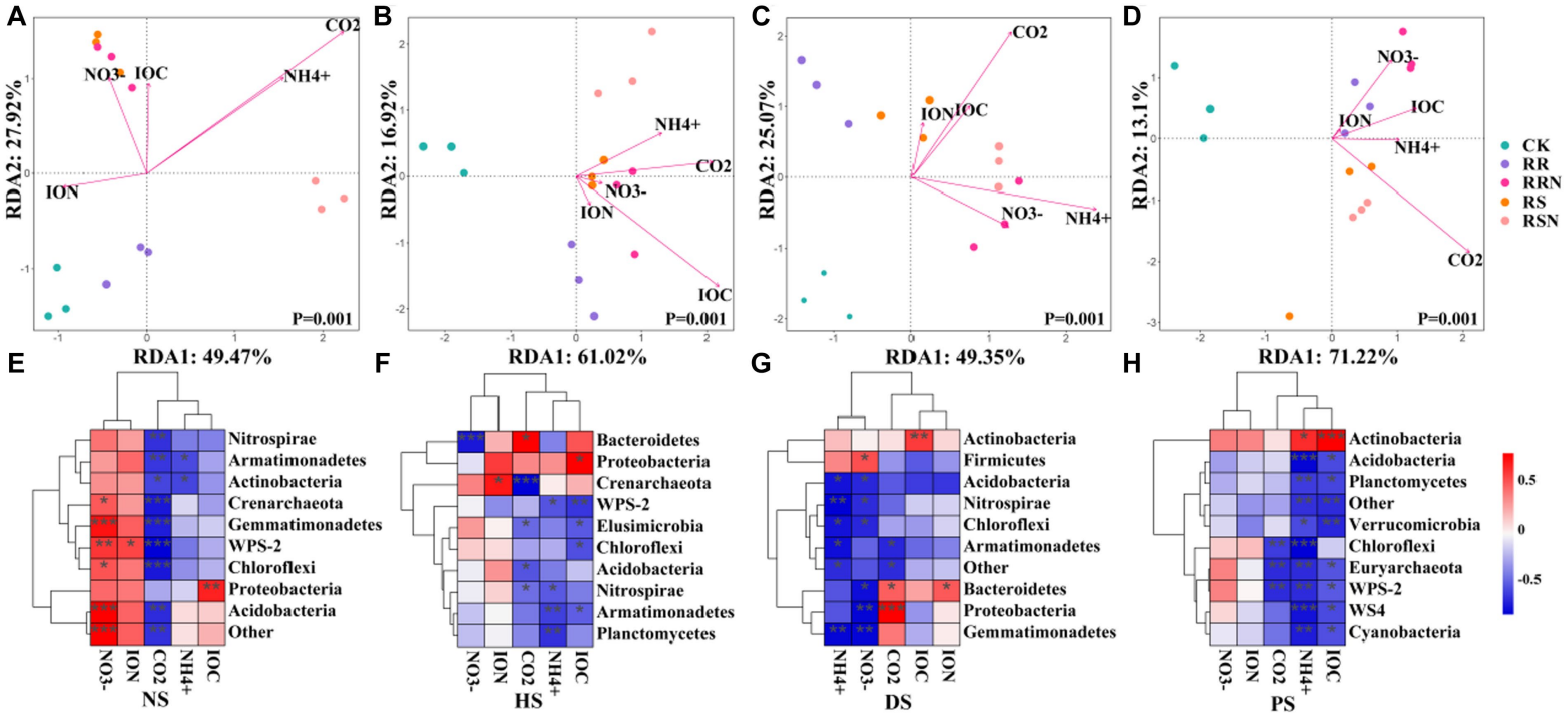
RDA analysis in natural forest soil **(A)**, healthy banana soil **(B)**, diseased banana soil **(C)**, paddy soil **(D)**. Relationships between bacterial community composition and soil properties in natural forest soil **(E)**, healthy banana soil **(F)**, diseased banana soil **(G)**, paddy soil **(H)**. Different letters indicate significant differences between different treatments (*p* < 0.05). Treatments: CK, no glucose and NH_4_NO_3_; RS, rice straw; RSN, rice straw and NH_4_NO_3_; RR, rice root; RRN, rice root and NH_4_NO_3_.

The results of redundancy analysis (RDA), coupled with soil microbial community structure and soil physicochemical data showed that microbial community structure differed significantly between various types of soil treatments (Figures 6A–D). In NS, the first and second RDA ranking axes explained 49.47 and 27.92% of soil microbial variability, respectively, indicating that soil physicochemical properties could explain 77.39% of soil microbial community differentiation (Figure 6A). In HS, the first and second RDA ranking axes explained 61.02 and 16.92% of soil microbial variability, respectively, indicating that soil physicochemical properties explained 77.94% of soil microbial differentiation (Figure 6B). In DS, the first and second RDA ranking explained 49.35 and 25.07% of the variation of soil microorganisms, respectively, indicating that soil physicochemical properties could explain 74.42% of soil microbial differentiation (Figure 6C). In PS, the first and second RDA ranking axes explained 71.22 and 13.10% of soil microbial variability, respectively, indicating that soil physicochemical properties could explain 84.32% of soil microbial differentiation (Figure 6D).

In NS, CK and RR microbial communities were mainly affected by insoluble organic nitrogen (ION), while other treatments were mainly affected by NH_4_^+^, NO_3_^−^, CO_2_ and insoluble organic carbon (IOC). In addition to the CK treatment, other types of soil had a greater influence on microbial community. CO_2_ was negatively correlated with bacterial phylum levels in NS (Figure 6E), NH4+ was negatively correlated with most bacterial phylum levels in DS and PS (Figures 6G,H), while physicochemical indices were less correlated with bacterial phylum levels in HS (Figure 6F).

### 3.7 Bacterial correlation network analysis and random forest analysis of bacterial community structure

Correlation network analysis revealed significant differences in carbon and nitrogen utilization by different types of soil microorganisms. In forest soil, the addition of rice straw and roots reduced the correlation of bacterial species compared with CK, while rice straw and nitrogen (RSN) significantly increased the correlation of bacteria (Supplementary Figure S4A). In healthy banana soil, the addition of rice straw and nitrogen (RSN) significantly reduced the correlation of bacteria (Supplementary Figure S4B). In the diseased banana soil, both rice root and rice root + nitrogen treatments significantly reduced the correlation of bacteria (Supplementary Figure S4C). However, in paddy soil, the addition of rice straw, roots and nitrogen significantly increased the correlation of bacteria compared with CK (Supplementary Figure S4D).

**Figure 7:**
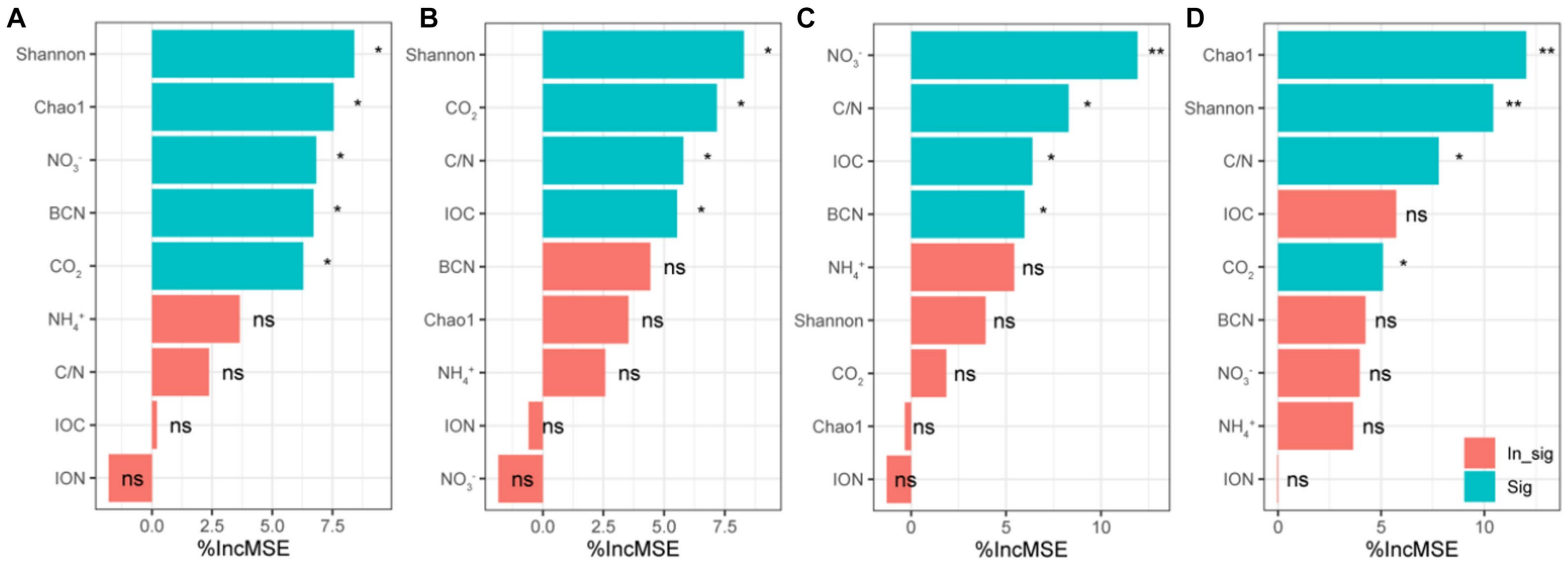
Random forest analysis of bacterial community structure (PCoA1) in natural forest soil **(A)**, healthy banana soil **(B)**, diseased banana soil **(C)**, paddy soil **(D)**. IOC: insoluble organic carbon; ION: insoluble organic nitrogen; BCN: bacterial gene copy number; CO2: carbon dioxide emissions; C/N: IOC/ION; NO_3_^−^: nitrate-N; NH_4_^+^: ammonium-N. ^*^: *p* < 0.05; ^**^ *p* < 0.01.

Through random forest analysis found that Shannon, Chao1, NO_3_^−^, BCN and CO_2_ were important predictors of bacterial community structure in forest soil (Figure 7A). In healthy banana soil, Shannon, C/N, IOC and CO_2_ were important predictors of bacterial community structure (Figure 7B). In the diseased banana soil, C/N, IOC, NO_3_^−^, BCN and NH_4_^+^ were important predictors of bacterial community structure (Figure 7C). In paddy soil, Shannon, Chao1, C/N and CO_2_ were important predictors of bacterial community structure (Figure 7D).

### 3.8 Linear regression analysis

**Figure 8:**
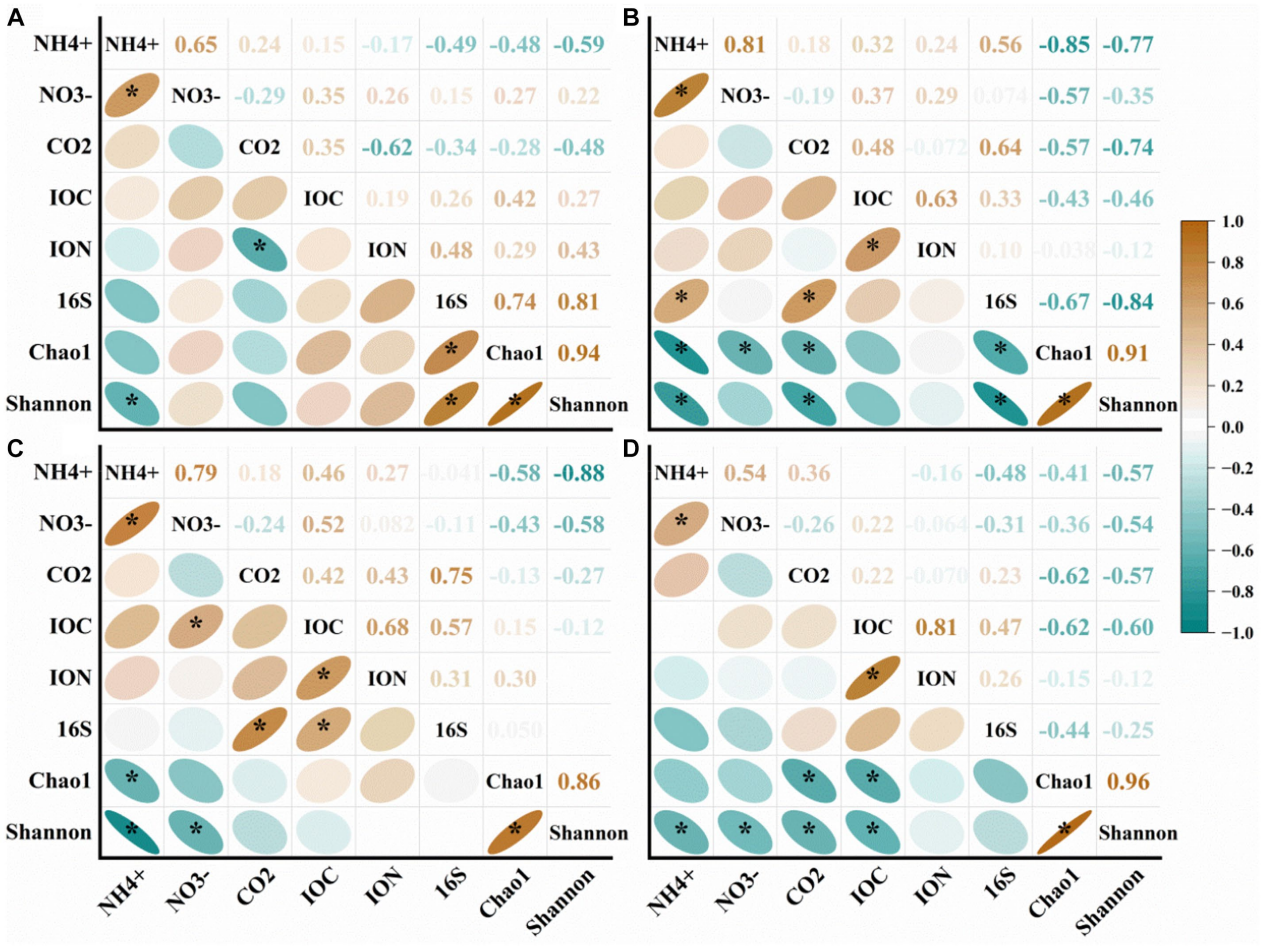
Correlation analysis of different types of soil properties in **(A)** natural forest soil (NS), **(B)** healthy banana soil (HS), **(C)** diseased banana soil (DS), **(D)** paddy soil (PS). IOC: insoluble organic carbon; ION: insoluble organic nitrogen; 16S: bacterial gene copy number; CO_2_: carbon dioxide emissions; NO_3_^−^: nitrate-N; NH_4_^+^: ammonium-N. ^*^: *p* < 0.05.

In our analysis, we observed a significant positive correlation between NH_4_^+^ and NO_3_^−^ in different types of soil (Figure 8). However, in the case of NS and HS, we found that 16S was correlated with Chao 1 and Shannon values (Figures 8A,B). In HS and DS, NH_4_^+^ showed a significant negative correlation with Chao 1 and Shannon values (Figures 8B,C). This suggests that an increase in ammonium nitrogen can lead to a reduction in both the diversity and abundance of soil bacteria, potentially compromising their ability to withstand environmental disturbances and increasing the likelihood of healthy banana soil transitioning to a diseased state. Furthermore, Shannon values showed a significant negative correlation with NH_4_^+^, NO_3_^−^, CO_2_ and IOC (Figure 8D).

## 4 Discussion

Land use practices determine plant community composition and soil management strategies, both of which affect soil physicochemical characteristics (Batlle-aguilar et al., 2011; Wolińska et al., 2017). Basic soil chemical properties were used as an indicator of nutrient content, and soil properties were obviously different across land utilization types. SOM plays a significant role in different ecosystem and soil fertility, determining the nutrient sequestration capacity of the soil (Dutta et al., 2003). Crop residues are well-known additives used to preserve and enhance soil fertility (Bhattacharyya et al., 2001) by sustaining or enhancing soil organic carbon (Li et al., 2018), which improves microbial activity and function, as well as plant biomass production and C sequestration (Jin et al., 2018). This study has showed that the addition of organic carbon (rice straw and root) along with inorganic nitrogen leads to an increase in the content of the soil insoluble organic C and SOC content. These findings generally consistent with the previous studies. A large body of results has found that below-ground C inputs (root biomass and root exudates) form stable carbon more efficiently than above-ground C inputs from litter (Cotrufo et al., 2015; Sokol et al., 2019; Sokol and Bradford, 2019; Witzgall et al., 2021). Moreover, our study found that the level of insoluble organic carbon in RR treatment was higher than that in the RS treatment. This suggests that belowground plant residues contributed a more significant contribution to SOC than aboveground biomass, a finding that is mostly consistent with previous studies.

The C metabolism characteristics of soil microbial communities can reveal the biological effectiveness and functional richness (Banning et al., 2012). Average CO_2_ production rate is often used to infer soil microbial activity and reveals the ability of soil microbial communities to use particular C sources (Sinsabaugh et al., 2013). Previous work has demonstrated that larger residue C inputs may drive higher rates of C sequestration (Clapp et al., 2000). It is widely acknowledged that soil respiration can demonstrate biological activity and decomposition of organic residue (Santos et al., 2012; Ferreira et al., 2014). A high microbial respiration rate might indicate high microbial productivity in an ecosystem (Islam and Weil, 2000). Here, we showed that the addition of a combination of straw and inorganic nitrogen increased microbial respiration and improved nutrient conversion efficiency, consistent with previous studies, but the effect of adding rice straw was more obvious. Studies have presented a mixed picture regarding the impact of nitrogen-fixing microorganisms. On one hand, nitrogen addition has been shown to reduce both the abundance and diversity of nitrogen-fixing microorganisms. However, it’s worth nothing that certain studies have highlighted that judicious nitrogen fertilizer application can actually improve the organic carbon content in the soil and increase and abundance of nitrogen-fixing bacteria, thereby bolstering the nitrogen-fixing capability of soil microorganisms. In some instances, studies have even indicated that the abundance and community composition of nitrogen-fixing bacteria remained largely unchanged when compared to the control group following nitrogen fertilization (Ogilvie et al., 2008; Orr et al., 2012). In this study, the nitrogen fixation potential of different types of soil showed a significant decrease after the nitrogen addition. This reduction was primarily manifested in a significant decrease in the abundance of ^15^N and *nifH* genes, which further confirmed that the addition nitrogen reduces the biological nitrogen fixation capacity of soil microorganisms.

Microbial diversity is considered to be a crucial component in global C cycle. However, bacterial communities are often overlooked in stoichiometric nutrient cycling studies (McGuire and Treseder, 2010; Graham et al., 2014). Changes in bacterial community and diversity also alter soil fertility, pH and other environmental conditions (Samaddar et al., 2018; Li et al., 2019). Zhaoxiang et al. (2020) suggested that bacterial community structure is strongly influenced by soil organic matter content and TN. In this study, the CK and RR microbial communities in NS were mainly affected by ION, while other treatments were mainly affected by NH_4_^+^, NO_3_^−^, CO_2_, and IOC. In addition to the CK treatment, other treatments’ effects on microbial community characteristics had a greater influence on other types of soil, indicating that soil physicochemical factors were an important factor regulating soil microbial community composition. The effect of these factors was different across soil types: IOC had an obvious influence on community composition under different treatments in HS, and NH_4_^+^ was a dominant factor in DS. Previous studies demonstrate that as microbial community composition changes in response to land use change, microbial functional diversity decreases. Agricultural intensification has been shown to change community composition to change microbial functional diversity. The reduction of functional diversity has been identified as a widespread trend as land-use intensification increases, and this has been associated with specific environmental conditions and bacterial groups (Benslama et al., 2021). Additionally, member of the order Actinomycetales are major contributors to carbohydrate and amino acid metabolism in differ environments (Verastegui et al., 2014; Xia et al., 2015; De la Cruz-Barrón et al., 2016). We found that, with the higher the soil use intensity (HS, DS, and PS), the lower the bacterial richness and diversity, this shows that tillage mode is one of the important factors affecting soil microorganisms, especially bacterial communities. And the relative abundance of different bacterial phyla in NS was quite different from other types of soils. In NS, the relative abundance of Firmicutes under RSN treatment was significantly higher than in other treatments. In HS and DS, the relative abundance of Actinobacteria was the highest, and it was lower under RSN treatment than in CK. In PS, each treatment type significantly increased the relative abundance of Actinobacteria to a greater extent than in other soil types, Therefore, the influence of actinomycetes in different types of soil is the most obvious.

In different ecosystems, soil microbes play an essential role in organic matter decomposition and nutrient cycles, thereby enhancing nutrient availability for plants. At the same time, microbes act as a sensitive indicator that can be used to predict future soil biological conditions and can be used to inform agricultural practices (Hartmann et al., 2015). Recent work found that SOC content and soil ecological environment were significantly improved through the application of crop residue treatments (Tang et al., 2019), which enhanced the soil environment and C and nutrient availability to support soil microbial proliferation. Additionally, adding SOM at regular intervals may enhance the level of microbial activities; improve carbon and nitrogen cycle, and soil microbial diversity (Van Bruggen et al., 2006). We found that the addition of RS to NS and RR to DS led to a significant increase in microbial diversity. This indicates that the microbial communities in natural forest soil and banana garden soil are more sensitive to the response of exogenous organic carbon. Previous findings have shown that soil microbial community diversity was higher with crop residue treatments (Frasier et al., 2016). PCoA showed significant differences in bacterial communities, who documented changes in microbial community composition under straw application (Bei et al., 2018). A recent long-term experiment found no significant effect of maize straw application and fertilization on bacterial community structure at the phylum level (Xia et al., 2019). Here, our results revealed that in natural forest soil, the addition of rice straw and roots had a significant effect on bacterial communities, the RSN group was clearly separated from other treatments. In healthy banana soil and diseased banana soil, the degree of separation between each treatment decreased, but in paddy soil, the CK treatment is clearly separated from the other treatments, the results showed that the effects of organic carbon addition on different soil bacteria types were significantly different, with the addition of nitrogen increased the degree of impact.

## 5 Conclusion

We have demonstrated that exogenous carbon and nitrogen addition significantly affected soil physicochemical properties, bacterial communities and biological nitrogen fixation. Specifically, the addition of rice straw increased the absorption and utilization of nitrate nitrogen by microorganisms. On the other hand, the addition of rice roots (RR) did not increase the absorption capacity of microorganisms to absorb inorganic nitrogen but does increase the content of poorly soluble organic carbon. Furthermore, it is important to note that the addition of rice straw increased microbial respiration and the utilization efficiency of N_2_ utilization. This showed that, across different soil utilization types, the addition of rice straw increases nitrogen fixation capacity by increasing the activity. Moreover, as soil types change, the number and variety of bacteria decrease progressively due to the addition of exogenous carbon (rice straw) and nitrogen, and microbial respiration intensity emerges as an important factor influencing the differences in the structure of bacterial communities. The addition of inorganic nitrogen further reduces bacterial diversity and richness, consequently reducing the ability of soil microorganisms to resist the environment challenges. In light of these findings, it is evident that the addition of rice straw and ammonium nitrate caused the accumulation of ammonium nitrogen in the soil environment, resulting in reduced bacterial diversity and richness. Therefore, we believe that for the implementation of soil nutrient retention strategies in agricultural practices while concurrently reducing the application of ammonium nitrogen. This approach is essential to maintain a healthy soil environment during agricultural production.

## Data availability statement

The datasets presented in this study can be found in online repositories. The names of the repository/repositories and accession number(s) can be found at: https://www.ncbi.nlm.nih.gov/, PRJNA906297.

## Author contributions

CH: Writing – original draft. KL: Methodology, Writing – original draft. JL: Resources, Writing – original draft. PF: Resources, Writing – original draft. YR: Writing – review & editing. ZJ: Writing – review & editing.
