# Supplementary material for: Rice straw increases microbial nitrogen fixation, bacterial and nifH genes abundance with the change of land use types
Source: Front Microbiol. 2024 Feb 28;14:1283675. doi: 10.3389/fmicb.2023.1283675 (PMC10933014; doi:10.3389/fmicb.2023.1283675)
Supplement: Supplementary file 1 [file Data_Sheet_1.docx]

**SUPPLEMENTARY MATERIAL**


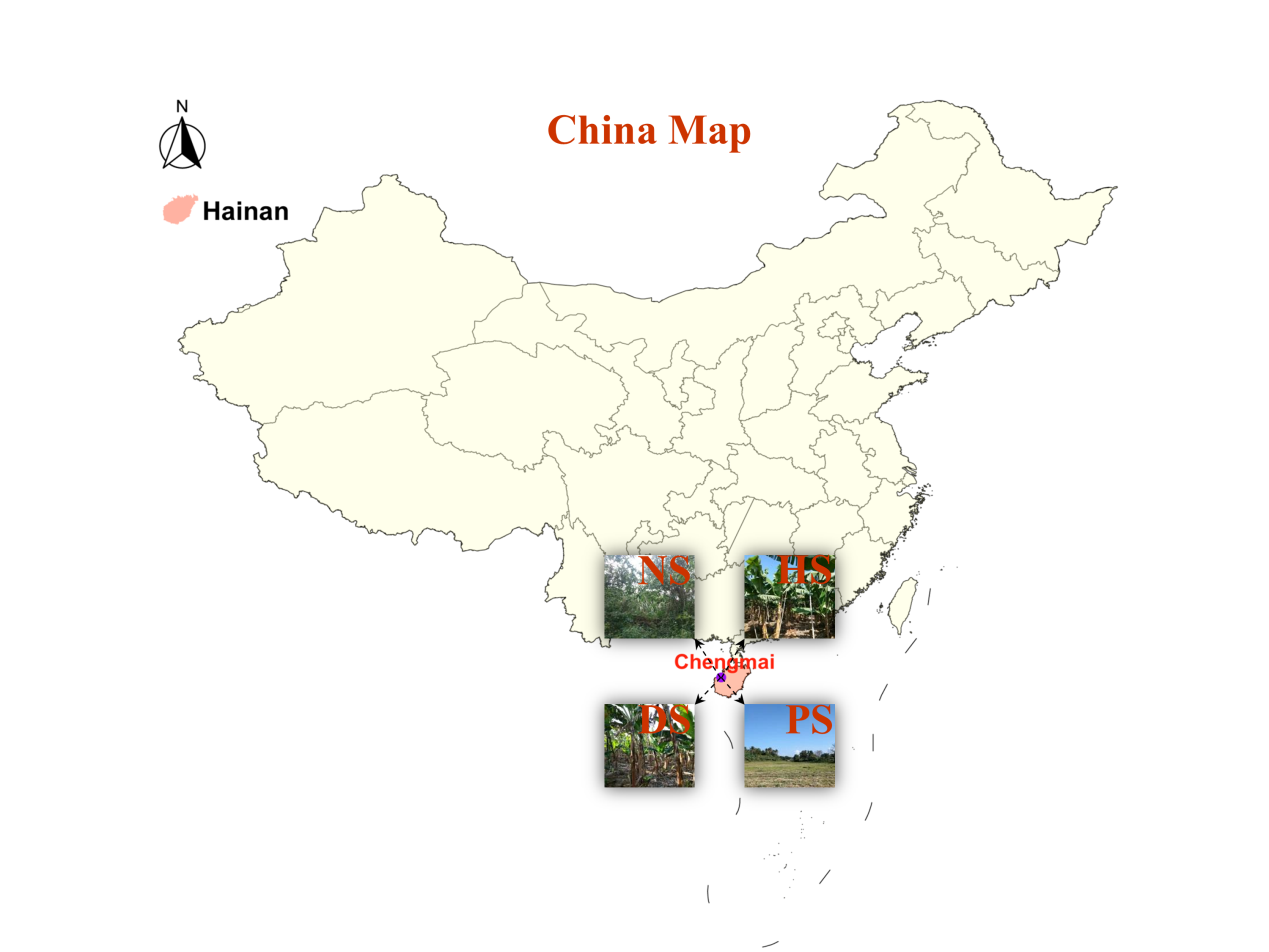


**Fig. 1** Distribution of soil sampling sites


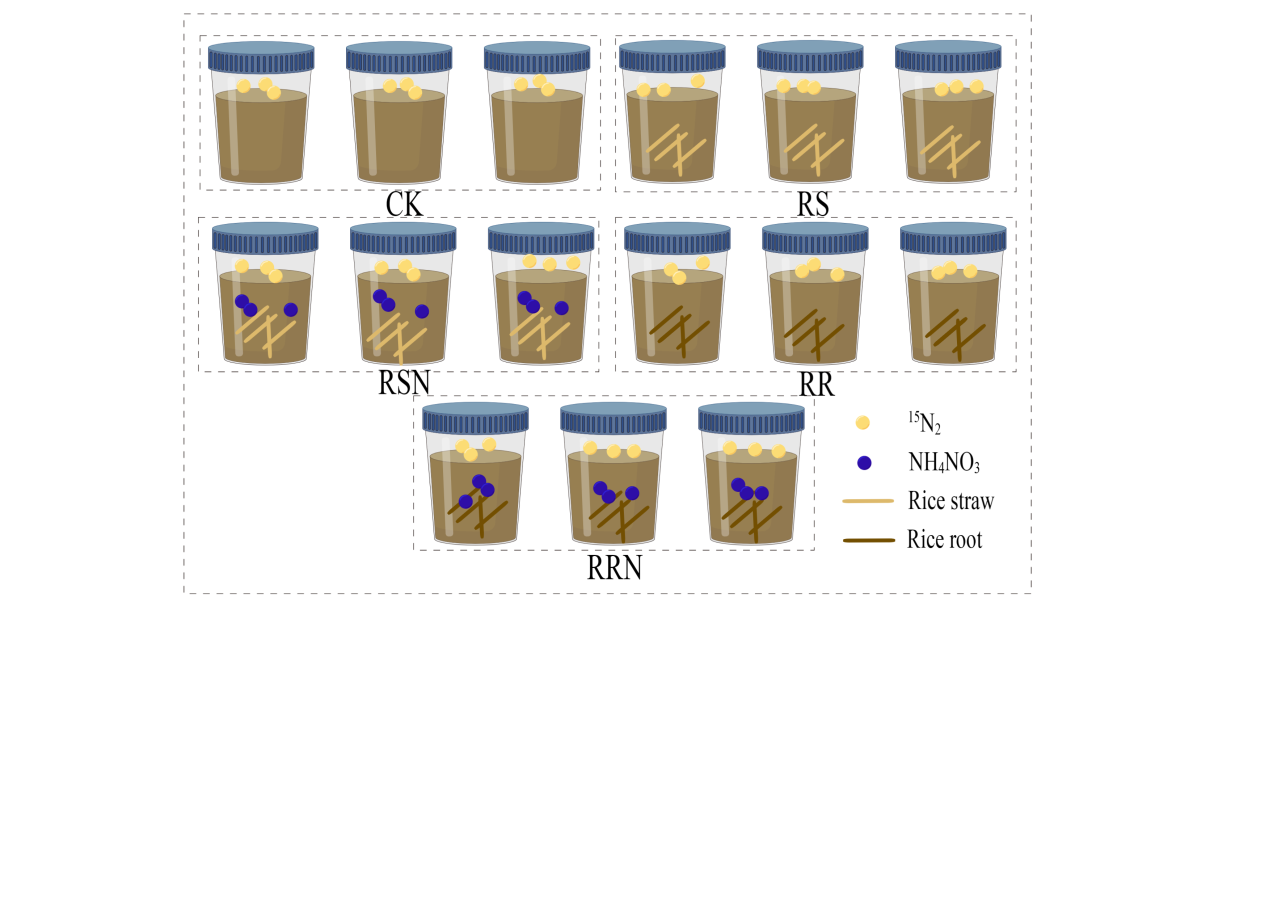


**Fig. 2** Schematic of the experimental design





**Fig. 3** Chao1 in natural forest soil (A), healthy banana soil (B), diseased banana soil (C), Paddy soil (D). Shannon in Natural forest soil (E), healthy banana soil (F), diseased banana soil (G), paddy soil (H). Different letters indicate significant differences across different treatments (p < 0.05). Treatments: CK, no glucose and NH_4_NO_3_; RS, rice straw; RSN, rice straw and NH_4_NO_3_; RR, rice root; RRN, rice root and NH_4_NO_3_.


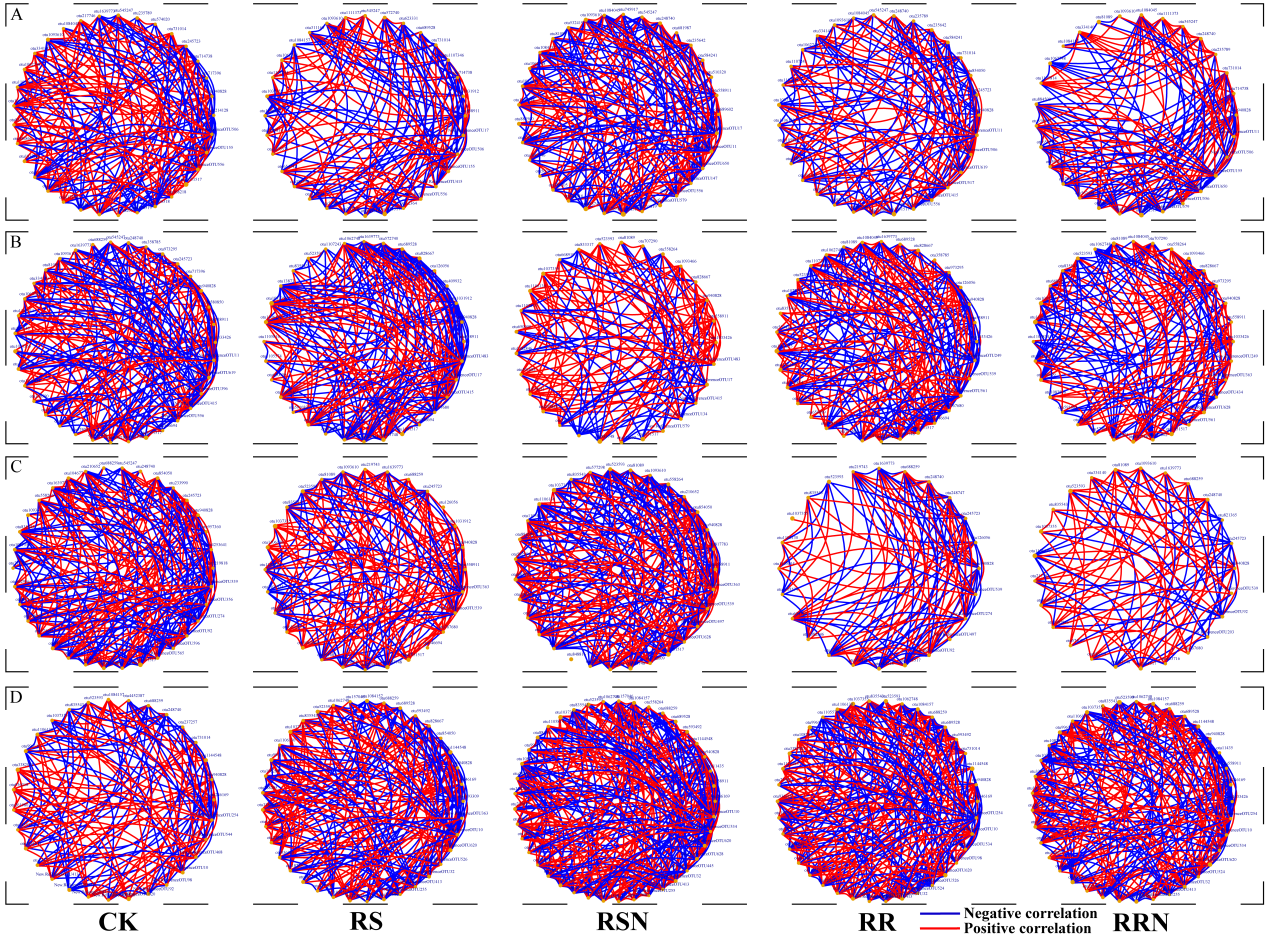


**Fig. 4** Bacterial related network analysis in natural forest soil (A), healthy banana soil (B), diseased banana soil (C), paddy soil (D). Treatments: CK, no glucose and NH_4_NO_3_; RS, rice straw; RSN, rice straw and NH_4_NO_3_; RR, rice root; RRN, rice root and NH_4_NO_3_.
